# Tumor microenvironment and exosomes in brain metastasis: Molecular mechanisms and clinical application

**DOI:** 10.3389/fonc.2022.983878

**Published:** 2022-10-20

**Authors:** Yirizhati Aili, Nuersimanguli Maimaitiming, Hu Qin, Wenyu Ji, Guofeng Fan, Zengliang Wang, Yongxin Wang

**Affiliations:** ^1^ Department of Neurosurgery, First Affiliated Hospital of Xinjiang Medical University, Urumqi, China; ^2^ Department of Four Comprehensive Internal Medicine, The First Affiliated Hospital of Xinjiang Medical University, Xinjiang, China; ^3^ School of Health Management, Xinjiang Medical University, Urumqi, China; ^4^ Department of Neurosurgery, Xinjiang Bazhou People’s Hospital, Xinjiang, China

**Keywords:** brain tumor, metastases, tumor cell, tumor microenvironment, exosomes

## Abstract

Metastasis is one of the important biological features of malignant tumors and one of the main factors responsible for poor prognosis. Although the widespread application of newer clinical technologies and their continuous development have significantly improved survival in patients with brain metastases, there is no uniform standard of care. More effective therapeutic measures are therefore needed to improve prognosis. Understanding the mechanisms of tumor cell colonization, growth, and invasion in the central nervous system is of particular importance for the prevention and treatment of brain metastases. This process can be plausibly explained by the “seed and soil” hypothesis, which essentially states that tumor cells can interact with various components of the central nervous system microenvironment to produce adaptive changes; it is this interaction that determines the development of brain metastases. As a novel form of intercellular communication, exosomes play a key role in the brain metastasis microenvironment and carry various bioactive molecules that regulate receptor cell activity. In this paper, we review the roles and prospects of brain metastatic tumor cells, the brain metastatic tumor microenvironment, and exosomes in the development and clinical management of brain metastases.

## Introduction

**Figure 1:**
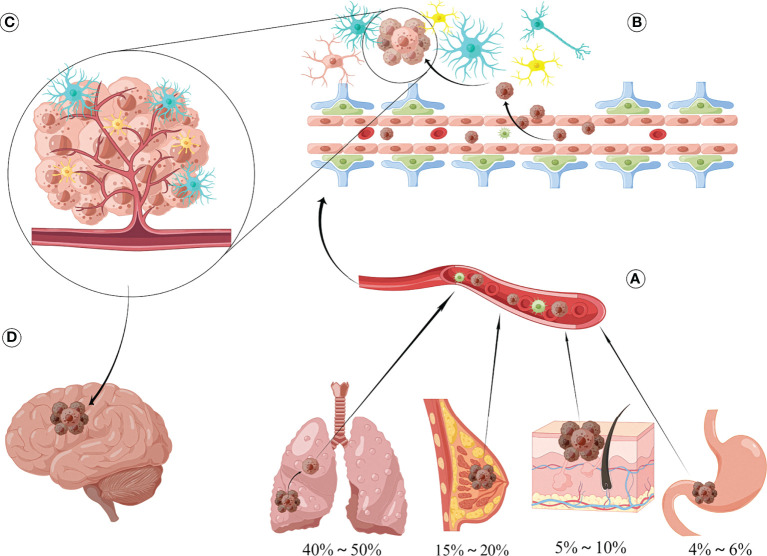
Sources and formation processes of brain metastases. **(A)** The most common sources of brain metastases are lung cancer (40%-50%), followed by breast cancer (15%-20%), skin cancer (mainly melanoma) accounting for 5%-10%, and gastrointestinal malignancy (4%-6%). **(B)** Tumor cells and secreted vesicle contents can disrupt the integrity of BBB, thereby promoting tumor metastasis to intracranial, interacting with surrounding astrocytes, microglia/macrophages, and then influencing the biological behavior of brain metastasis through various pathways such as secreting cytokine networks, direct contact and exosomes, and establishing complex networks. **(C)** Tumor cells can produce mutual adaptive changes with the components of the central nervous system microenvironment, and it is this interaction that determines the occurrence and development of brain metastatic lesions. **(D)** Metastatic foci appear in the skull, producing obvious mass and edematic effects, which seriously affects the quality of life of patients.

In the natural course, brain metastases occur in approximately 20–40% of patients with malignancies. Lung cancers are the most common source of brain metastases (40%–50%), and are followed by cancers of the breast (15%–20%), skin (mainly melanoma; 5%–10%), and gastrointestinal system (4%–6%) (**Figure 1**) (1). The survival and quality of life of patients with cancer have improved considerably in recent years due to the advent of newer treatment methods, and especially precision therapy. However, owing to the anatomical and physiological peculiarities of the central nervous system (CNS), it is difficult to achieve the desired effect of various treatments; brain metastases are therefore known as the last refuge of malignant tumors (2, 3). The median survival period in patients with untreated brain metastases is 1-2 months, while that of those who have been treated is approximately 6 months (4). The currently available treatments for brain metastases mainly include radiotherapy, systemic chemotherapy, and surgery. In this context, the widespread use of targeted drugs has improved the prognosis of these patients to a certain extent (5). However, the overall prognosis remains unsatisfactory.

Metastasis of tumor cells is one of the most important features of malignant tumors, and the intra- and inter-cellular molecular mechanisms involved in the metastasis process are considerably complex. These include epithelial-mesenchymal transition, survival of circulating tumor cells in blood vessels, tumor cell dormancy, and tumor cell heterogeneity and stemness, among others. The interaction between tumor and stromal cells, tumor-related angiogenesis, and a series of events related to the tumor microenvironment are also involved (6). The seed and soil hypothesis suggests that a specific tumor cell can only survive in a suitable tumor microenvironment; this explains the occurrence and development of tumor-specific metastasis (7). In this context, exosomes (a type of extracellular vesicles loaded with proteins, nucleic acids, and other signaling molecules) are involved in multiple processes leading to the development of brain metastases (8). Metastatic lesions to the CNS are unique compared to those in other organs (9). Evaluation of the biological characteristics of tumor cells that metastasize to the brain and their interaction with the microenvironment is therefore essential for the prevention and treatment of brain metastases.

## Proposal of the tumor microenvironment

The tumor microenvironment was first proposed in 1979, and has been variably termed as the tumor niche or tumor stem cell niche, among others (10). The tumor microenvironment refers to the local homeostatic milieu associated with tumorigenesis and metastasis, and is composed of tumor and non-tumor cells. It mainly includes tumor cells, tumor stem cells, endothelial cells, fibroblasts, immune cells, extracellular matrix structural components (such as collagen and elastin, among others) locally secreted cytokines, peptide growth factors, and other soluble substances (11, 12). It is widely accepted that the tumor microenvironment is a necessary functional unit for protecting and supporting tumorigenesis, development, metastasis, and recurrence; studies are also increasingly demonstrating the vital role of the tumor microenvironment in the evolution of tumors (13). Normal cells reside in a relatively stable internal environment (homeostatic milieu), and follow regulated processes for proliferation, differentiation, apoptosis, and the secretion and expression of related factors (14). Tumorigenesis involves a process that persistently disrupts this balance to alter the equilibrium in the local microenvironment, making it more suitable for tumor cell proliferation (15). Tumor cells proliferate indefinitely, and need to constantly shape an external tissue environment suitable for their growth. This involves the creation of tissue hypoxia and acidosis; formation of interstitial hypertension; and the production of a large number of growth factors, proteolytic enzymes, and immune inflammatory responses (16, 17). The tumor microenvironment provides shelter for metastatic tumor cells, protecting them from differentiation stimulation, apoptosis stimulation, and immune surveillance, thereby improving resistance to radiotherapy and chemotherapy (18). The tumor microenvironment can also induce tumor cell metastasis *via* secretion of cyclooxygenase-2 (COX2) and epidermal growth factor receptor (EGFR), the factors responsible for the organophilic nature of tumor cell metastasis. Tumor growth may therefore be inhibited by altering the local microenvironment of the tumor (19, 20).

## Characteristics of the microenvironment of brain metastases

The microenvironment of brain metastases has the following unique properties compared with that of other tissues (1): the presence of two biological barriers, namely, the blood-brain barrier (BBB) and blood-cerebrospinal fluid barrier (2), lack of immune cells such as lymphocytes and macrophages (microglia play an important role in the immune response) (3), lack of mesenchymal tissues such as fibroblasts (but is rich in astrocytes and oligodendrocytes), and (4) high expression of CNS-specific molecules such as CXCL-12 and neuroserpin (with neutropenia) (21–23). However, the role of the intracranial microenvironment in the development of brain metastases is debated. Previous studies have shown that tumor cells isolated from brain metastases models or co-cultured *in vitro* in the CNS microenvironment have stronger proliferation, invasion, and metastasis capabilities than the protocellular line (24). However, other studies have shown that astrocytes can secrete plasminogen activators to promote apoptosis of tumor cells; this is not conducive to tumor cell growth (**Figure 1**) (25). The impact of the intracranial microenvironment on tumor cells (based on the components that constitute the CNS microenvironment and their interaction with tumor cells) has been described below.

### Astrocytes

Astrocytes are the most abundant glial cells in the CNS. They are activated on stimulation, and appear morphologically hypertrophied; this is accompanied by increased expression of glial fibrillary acidic protein, a marker specific for astrocyte activation (26). Astrocytes perform a variety of functions, which include supporting nerve cells, nourishing nerve tissue, maintaining CNS homeostasis, forming a BBB, and repairing damaged CNS tissue (27). As the most important component of the CNS microenvironment, astrocytes play an important role in the formation of brain metastases (28). Following activation, astrocytes secrete a variety of cytokines that affect the proliferation, invasion, and metastatic ability of tumor cells (29). Studies have suggested that astrocytes can secrete matrix metalloproteinase (MMP)-2 and MMP-9, remove matrix components on the surface of tumor cells and the surrounding matrix, and promote the invasion and metastasis of tumor cells. In this context, MMP-2 and MMP-9 can activate transforming growth factor-β (TGF-β) (30), which in turn regulates cell growth, angiogenesis, and other functions through vascular endothelial growth factor (VEGF). Related clinical data show that patients with MMP-2-positive *in situ* or metastatic tumors of the brain have shorter survival times (31). A study on melanoma brain metastases found that astrocytes can produce interleukin (IL)-3, CD40L, CXCL 12, interferon-γ, and other cytokines that stimulate melanoma cells; in this context, IL-23 stimulates tumor cells to produce MMP-2, thereby promoting tumor cell proliferation (32). The mechanism of interaction between tumor cells and astrocytes (via cytokine networks) is considerably complex. Studies have shown that tumor cells in the CNS can secrete macrophage migration inhibitory factor, IL-8, and plasminogen activator inhibitor 1, thereby activating astrocytes; activated astrocytes can secrete IL-6 and tumor necrosis factor-α (TNF-α). TNF-α and IL-1β promote tumor cell growth; however, IL-6 receptor expression is down-regulated in these tumors (33, 34). However, as demonstrated by Sierra et al. (35), astrocytes also inhibit the growth of tumor cells. This is mainly mediated by fibrinolytic enzymes in the CNS that cause shedding of Fas ligand from astrocyte membranes; the secreted Fas ligand triggers apoptosis of tumor cells (36).

Astrocytes also protect tumor cells from the cytotoxic effects of chemotherapy. This protection may be mediated by direct contact with tumor cells and gap junctions (GJs); however, fibroblasts do not play a similar role (37–39). In this context, direct connexin 43-mediated intercellular communication between astrocytes and melanoma cells protects the latter against chemotherapy-induced apoptosis (40). Direct contact between astrocytes and tumor cells can also promote the secretion of IL-6 and IL-8 by tumor cells. Astrocytes then produce endothelin 1, which binds to the endothelin receptor of tumor cells to activate the AKT and mitogen-activated protein kinase pathways; this affects the downstream expression of Bcl-2-like protein 1, twist family basic helix-loop-helix transcription factor 1, and glutathione s-transferase alpha 5, thereby protecting the cells against chemotherapy drugs (41). Murphy et al. (42) found that connexin 43 can induce resistance to temozolomide by activating the AKT/AMP-activated protein kinase/mammalian target of rapamycin signaling pathway in malignant gliomas. However, studies have shown that connexin 43-mediated intercellular communication can enhance the cytotoxic effect of chemotherapy drugs in testicular cancer cells; in this context, studies suggest that GJs can transmit certain small molecules to induce tumor cell apoptosis (43). These findings suggest that specific GJ signaling molecules in tumor cells and the microenvironment can affect tumor cell sensitivity to chemotherapy in the CNS; they may block or activate signaling between GJs and offer clinically significant enhancement of chemotherapy drug effects.

As a key factor in the microenvironment, astrocytes can interact with tumor cells to influence the biological behavior of brain metastases *via* various channels such as cytokine networks and direct contact. The interaction between the two is complex and some mechanisms have not been fully understood; this needs further evaluation (44–46).

### Microglia/macrophages

Microglia play an important role in the CNS immune response (47). They belong to the monocyte-macrophage system, and it is difficult to distinguish them from circulating macrophages based on morphological and molecular markers after activation (48). Animal experiments have shown that in metastatic tumor models, microglia/macrophages are mostly derived from circulating monocytes; original intracranial microglia represent a minority. Certain studies therefore refer to activated microglia as microglia/macrophages (49).

The immune system plays an important role in tumorigenesis and development. Tumor macrophages can be divided into two types, namely, M1 and M2; the M2 mononuclear macrophage surface antigens CD163 and CD204 lead to secretion of arginase, IL-10, lipopolysaccharide, interferon γ, and transforming growth factor-β1. Cytokines such as transforming growth factor-β1 promote tumor growth. Conversely, M1 macrophages show high levels of inducible nitric oxide synthase expression and secrete IL-1, IL-12, nitric oxide, and TNF-α, all of which have tumoricidal effect (50). Wei et al. (48) studied the role of microglia/macrophages in glioma. They found that the M2 macrophages in the tumor microenvironment promoted glioma cell invasion, angiogenicity, and the formation of an inhibitory immune microenvironment, resulting in poor prognosis. Microglia/macrophages serve as the most important link for immune function in the CNS. It is therefore essential to identify the types of microglia/macrophages and the mechanisms of their production in brain metastatic tumors; this may help to confirm the relationship between the immune system and tumor cells in the CNS (51).

Data regarding the phenotypic changes of microglia/macrophages in brain metastases and the related mechanisms are lacking. Data regarding their impact on the treatment of tumors are also considerably scarce compared to those on current popular immunotherapy (52). *In vitro* experiments have shown that zoledronic acid can promote phenotypic changes in CNS microglia/macrophages to inhibit tumor invasion; clinical data also suggest that zoledronic acid can reduce the risk of recurrence in patients with breast cancer (53). However, sufficient clinical evidence is lacking for patients with brain metastases. Further trials are needed to evaluate the effect of microglia/macrophages in the treatment of brain metastasis (52, 54).

Recent research suggests that in addition to astrocytes and microglia, neurons and neurotransmitters play an important role in the occurrence and development of metastases (55). Zeng et al. (56) found that elevated N-methyl-D-aspartic acid (NMDA) receptor expression promotes the development of intracranial metastasis in breast cancer. The process is mediated by a protein subunit of the NMDA receptor, namely, GIuN2B, which is required for synapse formation and alteration of synaptic junction intensity; it is highly expressed in both human and mouse breast cancer cells. NMDA receptors allow calcium ions to enter the cells; this may be involved in the development of some human cancers. Zeng et al. (56) also found that human breast cancer cells express a protein known as neuroligin, which contributes to intercellular adhesion; it typically promotes the formation of synapes between neurons. This suggests that similar to human glioma cells, human breast cancer cells may exploit neuronal machinery to establish synaptic connections. Microscopy of mouse brain tissue samples containing human breast cancer cells has shown that the proteins that pack glutamate into vesicles are in close proximity to NMDA receptors; it also demonstrated the formation of synaptic structures between cancer cells and neurons. Compared with the mice that were injected with breast cancer cells having normal GIuN2B levels, the modified mice produced smaller brain tumors; they also had longer survival times with lower GIuN2B expression. This suggests that GIuN2B-mediated NMDA receptor signaling occurs through the formation of “pseudo” three-way synapses, which promote tumor cell colonization and growth in the brain. Several subsequent studies have shown that brain metastatic tumor cells can establish synaptic connections with neurons by using the molecular mechanisms involved in synapse formation between neurons. Synaptic activity causes depolarization in neurons and facilitates calcium ion flow, which is necessary for cell differentiation, proliferation, and survival. In cancer cells, this process promotes tumor colonization and progression (57, 58).

### Blood-brain barrier

The BBB is the structure with which tumor cells first come into contact during the development of brain metastases. It is composed of capillary endothelial cells and the tight connections between them, basal membranes, and dendrites of astrocytes (59). Under physiological circumstances, the BBB maintains CNS homeostasis and has an isolating effect on drugs, toxins, ions, and other substances (60). The tight connections of the BBB are the key to maintaining its integrity. These are composed of transmembrane proteins and surrounding proteins; the transmembrane proteins which constitute the connection between cells comprise occludin, junctional adhesion molecules, and the tight junction protein, claudin (mainly claudin-5 on BBB). The surrounding proteins are distributed on both sides of the tight junction (52, 61–63). Proteins such as the atresia band (zonula occludin [ZO]) and the filamentous actin-binding protein (afadin) maintain BBB stability (64). Animal experiments have shown that a variety of tumor cell lines can successfully pass through the BBB (65), and that the passage of tumor cells through the BBB is the first step in the formation of brain metastatic foci; however, the specific mechanism is not fully understood. On comparing differences in gene expression between brain metastatic lesions and protocellular cells, Bos et al. (66) found that COX2, α2,6-sialyltransferase (ST6Gal-I), and EGF can mediate passage of breast cancer cells through the BBB; they speculated that ST6Gal-I can specifically mediate brain metastasis by promoting acidification of endothelial cell surfaces (67). In patients with colon cancer, a single-nucleotide polymorphism of ST6Gal-I RS1736858 is highly associated with the risk of brain metastasis. The COX2 produced by tumor cells can induce the production of prostaglandins, which promote high expression of MMP-1 in tumor cells and degrade claudin and ZO-1 on the BBB (68). However, Lee et al. (69) suggested that the main source of COX2 was not the tumor cells, but the endothelial cells of the BBB. The neuropeptide, substance P, can also facilitate the passage of tumor cells through the BBB by changing the distribution and location of ZO-1 and claudin-5. *In vitro* studies have shown that small cell lung cancer cells can secrete placental growth factor after binding to VEGF-1 receptors, activate the extracellular signal-regulated kinase 1/2 pathway, promote occludin phosphorylation, and change the tight connections of the BBB, all of which eventually aid the easy transport of these cancer cells through the BBB (70).

Cell-secreted vesicle contents can also mediate tumor cell-induced destruction of the BBB. Studies have shown that miR-105 secreted by breast cancer cells can be transported across tight junctions *via* exosomes to destroy the integrity of the BBB; this promotes intracranial metastasis of tumor cells (71). However, some studies suggest that the destruction of the BBB does not only involve the ZO-1 tight junction protein. Tominaga and others found that breast cancer cell-secreted extracellular vesicles can be specifically taken up by endothelial cells of the BBB; miRNA-181c in the extracellular vesicles can inhibit the expression of phosphoinositide-dependent protein kinase 1 on endothelial cells of the BBB. Down-regulation of phosphoinositide-dependent protein kinase 1 can reduce actinin phosphorylation levels and activate cofilin; this causes conformational changes in actin, disrupts the tight connections of the BBB, and prompts breast cancer cells to pass through the BBB. Given the diversity and fragility of the mechanisms by which tumor cells cross the BBB, it may not be a good therapeutic target for resistance to tumor invasion (72, 73). Previous research on the mechanisms of tumor cell penetration of the BBB has mainly focused on breast cancer; studies on other cancers are relatively lacking. The presence of different mechanisms in various tumor cell types therefore warrants further exploration.

Another component of the BBB, namely, vascular endothelial cells, mainly interact with metastatic tumor cells by intercellular adhesion. In the early stages of brain metastasis in non-small cell lung cancer, tumor cells adhere with endothelial cells through VLA-4/VCAM-1, ALCAM/ALCAM, and LFA-1/ICAM-1 binding; these early adhesion molecules can therefore be used as targets to prevent brain metastasis (74). Other studies have shown that non-small cell lung cancer cells that metastasize to the brain have high levels of CD15 expression; they interact with TNF-α-activated CD62E on endothelial cells to mediate adhesion of tumor cells to microvessels (74). In addition, the interaction between tumor and endothelial cells can also promote tumor invasion and angiogenesis. Activation of the Janus kinase-signal transducer and activator of transcription pathway in tumor cells can cause them to secrete VEGF. In the vascular endothelium, this pathway is activated after VEGFR2 binding; this increases MMP-9 secretion and enhances the invasion ability of tumor cells (75).

The BBB limits antigen presentation and immune cell infiltration in the normal resting state. In order to enter the CNS parenchymal space in an inflammatory environment, T cells must first pass through the endothelial layer followed by the glial boundary (76). The vascular structure loses its integrity in patients with brain metastases, and may therefore promote other restrictions on the entry of peripheral immune cells. However, the more modern conceptual framework is that the BBB does not break down, but forms a blood-tumor barrier (BTB) instead; lymphocytes can pass through the intact BBB *via* the chemokine axis and multi-step adhesion process (77). In this context, the BTB has been shown to have heterogeneous permeability (regulated by reactive astrocytes); this may drive variable immune cell infiltration (78). The process of thrombotic inflammation, which has been recently studied in mouse models of acute stroke, provides new insights into the possibility of biological overlap between brain metastases and BBB/BTB immune interfaces. Studies have shown that clots form preferentially in cerebral microvasculature and tumor cells form large metastases at the site of stagnation in blood vessels; cancer cells embedded in the clot have a higher rate of successful extravasation (79). To date, minimal progress has been made on transformation strategies involving the development of BBB/BTB destruction methods, receptor agonists that alter permeability, radiosensitizing nanoparticles, and novel delivery platforms, all of which have been evaluated in phase I clinical trials (80). These focus areas for future research will not only require increased understanding on the BBB/BTB itself, but also specific knowledge of its role in regulating CNS anti-tumor immunity.

### Microvasculature in brain metastasis

Adequate blood supply is indispensable for tumor growth. The microvasculature therefore plays an important role in the metastasis and growth of tumor cells (81). Pathological findings from animal models of brain metastases have shown that tumor cells are mostly distributed around the microvasculature within a radial distance of 75 μm; tumor cells located 100 μm away from the microvasculature cannot not survive (82, 83). Kienast et al. (84) traced the fate of all tumor cells in a brain metastases model using fluorescence tracing; they found that the tumor cells that were separated from blood vessels had all died. In this context, Fidler et al. (85) found that the microvasculature of brain metastases has low microvessel density. However, the lumen is characterized by numerous abnormally dilated segments.

VEGF is a key factor in angiogenesis. Earlier experiments have shown that although it is necessary, its presence is not sufficient for the formation of brain metastases (86). Studies have shown VEGF levels in brain metastases tend to be higher than those of primary lesions; the levels also correlate positively with microvessel density (87). In addition to angiogenesis, VEGF can activate a proportion of dormant cells during brain metastasis, prompting proliferation to micrometastases (84). A retrospective analysis showed that the use of bevacizumab can effectively reduce the development of brain metastases in patients with lung cancer without increasing the risk of CNS bleeding (88). However, it should be noted that tumor vasculature formation is affected by many factors; the regulatory role of other factors therefore need to be considered (89).

### Other cellular components of the brain metastases microenvironment

Interactions of tumor cells with other cellular components such as oligodendrocytes, circulating immune cells, and CNS interstitial components have been less studied (90). Studies using natural killer cells in animal models of breast cancer or glioma showed that they inhibited the growth of glioma cells and human epidermal growth factor receptor (HER)-positive breast cancer cells. However, in animal models of breast cancer with brain metastases, CD11b-positive myeloid cells have been found to aggregate and form the “soil” for early tumor metastasis; this further releases the inflammatory factors S100A8 and S100A9, inducing tumor cell chemotaxis (91). Cancer associated fibroblasts have been found in human tumors of the CNS; research suggests that these fibroblasts promote tumor cell invasion (7).

## Biological characteristics of tumor cells in the brain metastases microenvironment

In the process of tumor metastasis, a series of biological changes occur in cells of distant metastatic foci to enable adaptation to the microenvironment (92–94). The alterations may manifest at the deoxyribonucleic acid (DNA) or epigenetic levels, thereby influencing phenotypic changes in tumor cells (**Figure 1**). The biological characteristics of brain metastatic tumor cells have been explained from the aspects of genetic alteration, post-translational modification, and metabolic characteristics of brain metastatic tumors (95).

### Gene changes in brain metastatic tumor cells

Brastianos et al. (96) examined 86 metastatic brain lesions and their matching primary lesions based on focal point mutations and copy number variations (CNVs), which map the evolutionary tree of tumor cells by calculating the individual cancer cell fraction. They estimated the homology between cells by measuring the number of gene copies near the mutation at the checkpoint, and found that although the tumor cells from the metastases and primary lesion originated from the same ancestor, they had different subclones. They also found homology between subclonal tumor cells from multiple intracranial foci. A series of other related studies (**Table 1**) have confirmed different genotypic changes such as single nucleotide variations, CNVs, deletion, and amplification, among others, between the primary and metastatic brain lesions. The changes mainly involve activation of multiple cell signaling pathways, apoptosis, and cell adhesion, and partially explain the mechanism of development of brain metastases (100, 101, 103, 104).

**Table 1:** Gene profile changes in brain metastasis tumor Microenvironment.

Study	Primary tumor(number)	Matched brain metastasis	Gene profiles	Implication
Sherise D.Ferguson et al. (97)	lung cancer (8178) Breast cancer(7064) Melanoma(757)	293 99 101	Mutation: RRM1,TS,ERCC1,TOPO1	DNA synthesis and repair and implicated in chemotherapy resistance
Brastianos PK et al. (94)	Lung cancer (38) Breast cancer (21) Renal carcinoma (10) Others (17)	15 12 3 8	Mutation : CDK,MLC1,HER2,EGFR,BRAF,MEK	Cell cycle proteins; PI3K/AKT/mTOR pathway; HER family; RAF/MEK/ERK pathway;
Bollig-Fischer et al. (98)	Breast cancer (10)	4	Amplification : HER2	HER family
Li F et al. (95)	Breast cancer(1)	1	CNV : Gain:Gain: 1p33-p34, 1q22, 5p13, 14q11 Loss:3p, 4q31, 5q, 11p15, Xp21-22, Xq21	CNV Gain: leukocyte migration and organ development; CNV Loss: proteolysis, negative regulation of cell proliferation and cell adhesion
Preusser M et al. (99)	Lung cancer(175)	175	Amplification : FGFR1	FGF/FGFR pathway;
Chen G et al. (96)	Melanoma (74)	30	CNV:generally identical BRAF,NRAF,CTNNB1 hot spot; Mutation : TP53;Loss : PTEN	CNV and hot spot mutations: generally identical
Lo Nigro C et al. (100)	Breast cancer (23)	23	Mutation : TP53	Anti-oncogene mutation
Wikman H et al. (37)	Breast cancer (128)	15	Loss : PTEN	PI3K/AKT/mTOR pathway
Ding L et al. (93)	Breast cancer (1)	1	WWTR1, SNV : NRK, PTPRJ,CNV:80.65% overlaps	SNV missense mutation; 19.35% of CNV difference
Gaedcke J et al. (101)	Breast cancer (102)	85	CNV : Gain:EGFR,HER2;Loss : ER,PR	HER pathway; Estrogen and progesterone receptors
Arai T et al. (103)	Lung cancer (11) Gastric cancer (9) Esophageal cancer (1) Breast cancer (1)	7 6 1 1	Amplification : HER2,EGFR	HER family

Studying the patterns of change and the mechanisms by which they arise may provide promising therapeutic targets for brain metastases (99). In this context, a study on 86 patients with breast cancer showed the presence of clinically relevant therapeutic mutations in metastatic lesions from the brain; these included mutations of HER2, EGFR, the B-Raf proto-oncogene, and AKT. These genes were not detected in the primary lesion. The CNVs of HER1 and HER2 were higher in the metastatic brain lesions than in the primary lesion; however, the CNVs of hormone receptors including the estrogen and progesterone receptors had decreased (105). Genetic alterations such as fibroblast growth factor receptor amplification and B-Raf proto-oncogene and neuroblastoma RAS hotspot mutations can also be detected in brain metastases (**Table 1**) (98, 106).

### Epigenetic changes in the brain metastases microenvironment

A study had compared the whole genome methylation levels of tumor cells in brain metastases of nude mice with those of subcutaneous tumor models of melanoma, lung cancer, stomach cancer, and other cell lines; the methylation levels of a series of transcription factors such as transcription factor 4, purine rich element binding protein B, one cut homeobox 2, estrogen related receptor gamma, nuclear factor IB, and myocyte enhancer factor 2C were found to differ significantly in the brain metastases model. In particular, the difference in transcription factor 4, a transcription factor related to neurodevelopment, was the most obvious (107). Tumor cells that metastasize to the brain have unique gene expression profiles owing to these changes (108). Marzese et al. (109) also observed an inconsistency in methylation levels between brain metastases and extracranial lesions of human melanoma; methylation levels were significantly increased in the promoter range of the homeobox A9 gene (among members of the homeobox family), a transcription component that encodes multiple genes and induces changes in neuro development-related genes (110). In a study on breast cancer, the methylation levels of genes such as polypeptide N-acetylgalactosaminyltransferase 9, coiled-coil domain containing 8, and basonuclin 1 were significantly higher in brain metastases than in the primary lesions; *in vitro* silencing of the mentioned genes could enhance the invasion ability of tumor cells (**Table 1**) (111). Changes in methylation levels of tumor cells in the CNS may be responsible for changes in tumor phenotype; however, the mechanism for the changes is not fully understood.

Recent studies have confirmed that micro ribonucleic acids (miRNAs) are one of the key factors affecting protein expression after transcription (112). By comparing miRNA levels between the primary lesion and brain metastases, Zhao et al. (113) identified a group of down-regulated miRNAs in patients with lung cancer; these included miR-145, miR-214, miR-9, and miR-1471. Among these, miR-145 was the most obviously down-regulated. In this context, the down-regulation of miR-145 may promote the proliferation of the A549 and SPC-A1 cell lines in lung cancer. Previous studies have shown that miR-145 can affect the proliferation and invasion of lung cancer cells by participating in the regulation of c-Myc, EGFR, and nudix hydrolase 1 expression (114). MiR-145-5p, another member of the miR-145 family, was also found to be significantly down-regulated in patients with brain metastases from lung cancer; this increased the expression levels of downstream EGFR, octamer-binding transcription factor 4, mucin 1, c-Myc, and tumor protein D52. In this context, the down-regulation of miR-145-5p was caused by initiation of interval methylation (115). MiR-141-3p and miR-200b-3p of the miR-200 family have also been found to be significantly up-regulated in metastatic brain lesions than in primary tumors; they down-regulate zinc finger E-Box binding homeobox 2 expression, thereby affecting the proliferation and invasion ability of tumor cells (116).

### Characteristics of tumor cell metabolism in the microenvironment of brain metastases

The CNS has an abundant blood supply, with a blood flow that accounts for 1/5 of the total body volume; blood and energy supplies to the CNS are therefore relatively sufficient (117). Due to the presence of the BBB, the levels of glucose in the interstitial fluid of the CNS are lower than those in the blood. However, it has abundant levels of branched-chain amino acids such as leucine, valine, isoleucine, and glutamic acid (118).

Compared to the invasion and proliferation characteristics of brain metastases, the metabolic characteristics of tumor cells in the CNS have been relatively underexplored (119). Chen et al. (120) compared the levels of protein expression related to energy metabolism between tumor cells in brain and bone metastasis models; they found that unlike common tumor cells which rely predominantly on anaerobic metabolism, tumor cells in the CNS actively demonstrate tricarboxylic acid cycle-oxidative phosphorylation with activation of the pentose phosphate pathway. This may induce resistance of tumor cells to certain antimetabolite chemotherapy drugs such as D-2-deoxyglucose (121). Chen et al. (122) found that breast cancer cells that metastasize to the brain have greater tolerance to low sugar levels than their parent cells; they also express more glutamate dehydrogenase and α-ketoacid dehydrogenase to use the glutamic acid and branched-chain amino acids available in the environment.

In terms of lipid metabolism, Chen et al. (120) found fatty acid-β oxidation-related enzyme profile expression to be higher in the animal brain metastases model than in the bone metastases model. However, the human breast cancer brain metastases tissue chip showed the expression levels of acetyl-CoA oxidase-1 and fatty acid synthase to be higher than those of metastases to other sites. This suggests that the processes of lipid synthesis and catabolism were more active in the metastatic brain lesions (123).

### Tumor cells in the brain metastases microenvironment acquire nerve cell properties

Park et al. (107) found that tumor cells in animal models of brain metastasis from lung cancer, melanoma, and colon cancer showed certain characteristics of neuronal cells; the levels of glutamate signaling pathway proteins and neurotransmitter complex proteins such as synaptosomal-associated protein 25 and synaptosomal-associated protein 91 were significantly increased. Similarly, brain metastases models of human breast cancer showed the expression of γ-aminobutyric acid (GABA) receptors and transaminase sources to have increased; this allows tumor cells to use the available GABA in the CNS for various metabolic activities (124, 125). Nygaard et al. (126) found the expression of glutamate-related signaling pathway signaling proteins, glutamate receptor ionotropic AMPA 2 and glutamate metabotropic receptor 4, to have increased in patients with melanoma brain metastases and animal models; this promotes the growth of tumor cells. Studies have also shown that plasmin found in the CNS can induce apoptosis of tumor cells; however, breast and lung cancer cell lines highly express neuroserpin, a neuronal inhibitor of plasminogen activator, thereby evading the pro-apoptotic effect of plasmin (127). This finding may be based on the fact that high levels of chloride in the interstitial fluid have a damaging effect on non-neuronal cells; coupled with the abundant neurotrophic factors, glutamate, and other substances in the CNS, this may induce certain neuronal properties in tumor cells to make them more suitable for survival in the CNS microenvironment (128).

## Role of exosomes in the brain metastases microenvironment

Exosomes are membranous vesicles with a diameter of between 30-100 nm; they have a lipid bilayer; can be secreted from all kinds of cells; are present in serum, urine, saliva, and other human body fluids; and can be separated and purified by ultracentrifugation, density gradient centrifugation, and other methods (129). Exosomes have a considerably complex composition, and include a variety of lipids, proteins, mRNAs, miRNAs, long non-coding RNAs, circular RNAs, and DNA. Advances in exosome-related research in recent years is gradually revealing the causes for organ propensity of metastatic tumors (102). Hoshino et al. (130) found that tumor exosome integrin expression profiles determine the organ propensity of tumor metastasis; they also found that ingestion of exosomes in the brain can create a pre-metastatic microenvironment for tumor metastasis. This suggests that exosomes can alter the pre-metastatic microenvironment to help target tumor localization (**Figure 1**). Studies also suggest that CD46 found on endothelial cells of human cerebral microvasculature is a receptor that mediates melanoma exosome uptake; this further confirms the role of exosomes in tumor targeting (131). Exosomes can also help target the localization of tumors by altering the manner by which energy is metabolized. Fong et al. (130) found that cancer cells inhibit glucose uptake by non-tumor cells; they down-regulate pyruvate kinase, and thereby glycolysis, in the pre-metastatic microenvironment by secreting high levels of exosomal miR-122. This suggests that exosomes can also promote tumor brain metastasis by changing glucose uptake in the pre-metastatic microenvironment (**Table 2**) (134).

**Table 2:** The role of exosomes in brain metastasis.

Study	Exosomal original	Exosomal cargo	Role in brain metastatic process
Umeze et al. (132)	Multiple myeloma	miR-135b	promotes neoangiogenesis
Fong et al. (130)	Breast cancer	miR-122	Reduces glucose uptake in normal brain cells
Wu et al. (133)	Non-small cell lung cancer	Lnc-MMP2-2	Destroys the tight junctions of the BBB
Tominaga et al. (72)	Breast cancer	miR-181c	Destroys the BBB by modulating the actin dynamics
Zhang et al. (134)	Normal astrocytic cells	PTEN targeting miR-19a	Reduces PTEN expression in brain metastatic tumor cells
Lu et al. (65)	Breast cancer	Lnc GS1-600G8.5	Disrupts the BBB by targeting the tight junction proteins
Zhou et al. (133)	Breast cancer	miR-105	Destroys the endothelial cell barrier by down-regulating ZO-1 tight junctions
Satelli et al. (135)	Lung cancer	Vimentin	promotes vimentin expression in the brain metastatic and induces EMT
Xing et al. (136)	Breast cancer	miR-503	Induces the release of tumoral growth factors and microglial reprograming leading to immune suppression microenvironment
Zhi et al. (137)	Lung cancer	S100A16	Improves the survival of SCLC metastatic cells in cerebrum
Rodrigues et al. (138)	Lung and breast cancer	CEMIP	Induces a proinflammatory vascular niche,promoting metastasis
Puigdelloses et al. (139)	Lung cancer, breast cancer, colorectal cancer, melanoma, pancreatic cancer, gastroesophageal cancer, bladder cancer	RNU6-1	Regulates tumoral growth rate

### The role of exosomes in the proliferation of brain metastases

#### Stromal cell exosomes promote the proliferation of brain metastases

**Figure 2:**
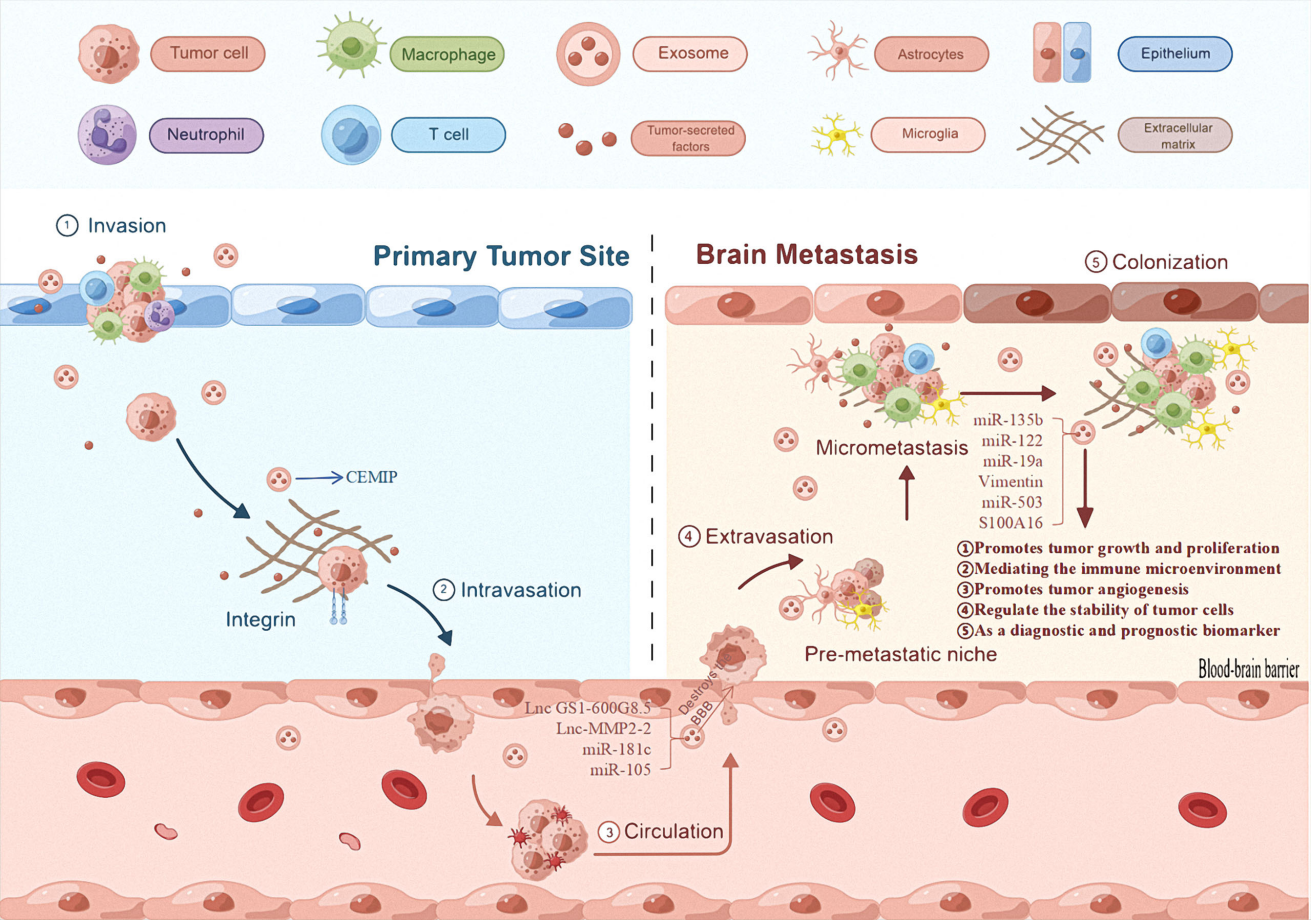
The role of exosomes in brain metastases. The first mechanism of primary tumor exosomes is that they can promote their own progression and metastasis. The second general mechanism is that exosomes derived from primary tumor cells promote the proliferation of brain metastases, regulated immune mechanism to promote tumor cell proliferation, and regulate the stability of tumor cells, and can be useful diagnostic and/or prognostic biomarkers.

Phosphatase and Tensin homolog deleted on chromosome ten is a tumor suppressor gene with phosphorylation activity, that regulates the apoptosis and proliferation of tumor cells (140). Zhang et al. (141) found that exosomal miRNA-19 produced by astrocytes can target the inhibition of Phosphatase and Tensin homolog tumor suppressor genes, resulting in increased secretion of chemokine ligand-2 and nuclear factor kappa-B; this promotes the growth of brain metastases. This shows that stromal cell exosomes found in the microenvironment can promote tumor cell growth by carrying miRNAs to influence tumor cell proliferation and inhibit apoptosis (**Table 2**; **Figure 2**).

#### Exosome-regulated immune mechanisms promote tumor cell proliferation

Metastatic exosomes can change the microenvironment to promote tumor cell proliferation (142). A study on breast cancer brain metastases found that X-inactive specific transcript deletion increased the secretion of exosomal miR-503, which was transmitted to microglia; this led to M1-M2 transformation and inhibition of T cell proliferation, enabling tumor immune evasion (136, 137). This confirms that tumor cell exosomes can regulate immune cells to enable tumor cell immune escape mechanisms and provide conditions for tumor cell proliferation (**Table 2**; **Figure 2**).

#### Exosomes regulate the stability of tumor cells

A variety of apoptotic mechanisms are often accompanied by a decrease in mitochondrial membrane potential (143). Xu et al. (144) found that exosomes can prevent the loss of mitochondrial membrane potentials through the prohibitin 1 protein present on mitochondrial membranes; tumor cells can therefore tolerate apoptosis in a stressed environment. This indicates that exosomes can regulate tumor cell stability and promote tumor cell proliferation by influencing mitochondrial membrane potential (**Figure 2**).

### Diagnostic significance of exosomes in brain metastases

Liquid biopsy technologies are rapidly gaining attention because of their rapid and non-invasive characteristics. Common techniques for tumor fluid biopsy include traditional circulating tumor cell detection, circulating tumor DNA detection, and tumor cell exosome detection (144, 145).

#### Advantages of exosomes in diagnosis

Exosome-based diagnosis offers the following advantages: (1) exosomes exist in almost all body fluids, are easier to enrich, and are more sensitive to current detection methods, and (2) they have high stability, allowing a large number of specific proteins to be isolated at temperatures as low as -80°C. However, exosome-related research has started recently and there is a paucity of cumulative data from studies; further in-depth research and analysis is required (146).

#### Prospects of exosomes in the diagnosis of brain metastases

In recent years, studies have focused on the role of exosomes in brain metastases (147). Camacho et al. studied the miRNA and protein profiles of brain metastasis-competent exosomes (148). Multiple proteins pertaining to cell communication, the cell cycle, and key signaling pathways involved in cell invasion and metastasis are promising biomarkers for brain metastases. Although exosomes are still in their infancy as biomarkers for the diagnosis of brain metastases, they are already in use for the diagnosis of lung cancer. Exosome Diagnostics made a major breakthrough in 2016 with the ExoDx™ Lung (ALK) technology for detection of exosomal miRNA by analysis of blood samples; this is being widely used. Combining exosomal miRNA and circulating tumor DNA analysis can increase diagnostic sensitivity by approximately 3-fold compared to circulating tumor DNA-based diagnosis alone (149).

#### Exosomes can be used as targets and tools for the treatment of brain metastases

Exosomes are involved in almost all processes of brain metastasis including cell genesis, metastasis, and proliferation. It is possible to target exosomes and the corresponding nucleic acids and proteins to treat the corresponding tumors; this provides new concepts for the treatment of brain metastases (150). In a recent study, Yang et al. (151) found that exosomes secreted by tumor cells contain functional programmed death-1 (PD-L1) protein, which can be transferred to other cells to inhibit T-cell resistance; binding of PD-1 to T cells inhibits anti-tumor immunity and protects the tumor cells. Inhibiting the secretion of PD-L1-containing exosomes by knocking down Rab27a or applying the inhibitor GW4869 can lead to meaningful anti-cancer effects. This offers a major step towards precision and individualized treatment of brain metastases (152).

The BBB has always been the greatest challenge in the treatment of brain metastases. Most traditional chemotherapeutic drugs and large molecule targeted drugs are denied entry by the BBB, thus making the CNS a sanctuary for survival and multiplication of metastatic cells (153). As a drug delivery system, exosomes may effectively address the issue BBB permeability to chemotherapy drugs (154). There are two main types of drug delivery methods for exosomes: exogenous and endogenous (155). Exogenous drug delivery requires the extraction of exosomes from donor cells and the delivery of small molecules (paclitaxel, adriamycin, and curcumin, among others) or gene-based drugs (e.g., small interfering RNA) into the exosome by electroporation (156). The endogenous drug delivery method carries the drug out of the cell *via* exosome secretion after the drug enters the donor cell; the drug-loaded exosome is finally extracted (157). Reversible protein-protein interaction molecules have been designed for large molecule proteins; these are controlled by blue light to allow integration into the endogenous pathway of exosome production, and are successfully loaded as “cargo proteins” into the new exosomes. This provides an important method for carriage of large molecule proteins by exosomes (158). Yang et al. (159) reported that exosomes derived from endothelial cells of mouse brain microvasculature can effectively deliver antitumor drugs *in vivo* across the BBB to inhibit tumor growth. This is indicates the advantages and possibilities of exosome treatment for brain metastases.

## Discussion

Changes in the biological properties of brain metastatic tumor cells and the interaction between tumor cells and their microenvironment may explain the relatively inefficient metastatic process of tumor cell colonization and growth in the intracranial “soil.” However, it is unclear whether the cranial microenvironment plays a screening or inducing role in the altered biological behavior of tumor cells in metastatic lesions (160). A study that examined genome-wide methylation levels of lung cancer and melanoma cells co-cultured with astrocytes partially replicated the altered methylation profile in animal models of brain metastasis; this suggests that astrocytes can cause changes in methylation levels in tumor cells (161). However, McDermott (162) proposed a model in which CNS microenvironment components such as astrocytes and microglia interacted with tumor cells to produce certain cytokines; these cytokines altered miRNA levels in tumor cells, which in turn affected the expression of the corresponding target genes.

Several studies have shown that tumor cell exosomes are closely related to tumor metastasis. The alteration of the cranial microenvironment and targeted migration of cancer cell exosomes are particularly important for the development of lung cancer brain metastases. In this context, exosomes play an important role in the tumor microenvironment and are directly or indirectly involved in intercellular signal transduction, tumorigenesis, and progression in the tumor microenvironment (163). For example, exosomes secreted by lung cancer cells contain oncogenes and they target the tumor microenvironment, thus promoting tumor progression (164). Exosomes are involved in DNA methylation, histone modification, post-transcriptional regulation, and RNA regulation. The relevant substances delivered by exosomes reflect the state of the cell, and exosomes originating from tumor cells may alter the tumor and promote the expression of tumor suppressor genes in recipient cells. Thus, exosomes in body fluids (including blood) may serve as biomarkers of cancer, and the detection of these biomarkers may be used for diagnosis or prognostic assessment of cancer.

As a target system, exosomes are expected to effectively promote the development of medical oncology. The relationship between exosomes and brain metastases needs to be explored further to understand the intrinsic mechanisms of exosome structure and their interactions with regulatory proteins (165–167). Along with the in-depth study of exosomes in brain metastases, their monitoring can aid the screening of susceptible or high-risk groups, clinical diagnosis, molecular staging, prognosis assessment, recurrence or metastasis prediction, and efficacy evaluation. In particular, the monitoring of exosomes may aid the formulation of brain metastases prevention strategies and the establishment of a risk evaluation system.

## Conclusion

In conclusion, the metastatic tumor microenvironment is a complex biological system. The mechanisms of metastasis formation and regulation that are associated with the microenvironment are areas of particular interest in cancer research. Findings indicate that the factors in the microenvironment that promote the formation of metastases are interrelated and interdependent. The proposed “seed and soil” hypothesis provides a broad framework for addressing the growth of brain metastases. As metastasis is almost always closely related to the formation and alteration of the microenvironment in a large number of cancers, continuous research on the microenvironment will improve understanding on the mechanisms of metastasis development and provide new targets for their diagnosis and treatment. Finally, as new therapeutic tools, exosomes are expected to be ideal markers for the early diagnosis of brain metastases and new targets for their treatment.

## Author contributions

ZW and YW designed the study; YA and NM wrote the first draft of the manuscript; HQ and WJ prepared the figure; GF prepared the Tables. All authors approved the final version of the manuscript for submission.

## Funding

This study was supported by The Xinjiang Uygur Autonomous Regional Cooperative Innovation ProgramGrant (NO. 2021D01A02), Regional Collaborative Innovation Special Project of Xinjiang Uygur Autonomous Region (No.2021E01013).

## Conflict of interest

The authors declare that the research was conducted in the absence of any commercial or financial relationships that could be construed as a potential conflict of interest.

## Publisher’s note

All claims expressed in this article are solely those of the authors and do not necessarily represent those of their affiliated organizations, or those of the publisher, the editors and the reviewers. Any product that may be evaluated in this article, or claim that may be made by its manufacturer, is not guaranteed or endorsed by the publisher.
